# Fate of Benzophenone, Benzophenone-3 and Caffeine in Lab-Scale Direct River Water Treatment by Hybrid Processes

**DOI:** 10.3390/ijerph18168691

**Published:** 2021-08-17

**Authors:** Minja Bogunović, Tijana Marjanović, Ivana Ivančev-Tumbas

**Affiliations:** Department of Chemistry, Faculty of Sciences, Biochemistry and Environmental Protection, University of Novi Sad, Trg. Dositeja Obradovića 3, 21000 Novi Sad, Serbia; tijanam@dh.uns.ac.rs (T.M.); ivana.ivancev-tumbas@dh.uns.ac.rs (I.I.-T.)

**Keywords:** drinking water treatments, emerging contaminants, ultrafiltration, activated carbon, coagulation

## Abstract

Emerging microcontaminants benzophenone (BP), benzophenone-3 (BP-3) and caffeine (CF) are widely used anthropogenic markers from a group of pharmaceuticals and personal care products. They have different logD values and charges at neutral pH (2.96 neutral for BP; 3.65 negative and neutral for BP-3; 0.28 and neutral for CF). The goal of this study was to assess the efficacy of coagulation/flocculation/sedimentation (C/F/S), adsorption onto two types of powdered activated carbon (PAC)/sedimentation (PAC/S) and the combination of these two processes in different dosing sequences (PAC/C/F/S) and with/without ultrafiltration (powdered activated carbon/ultrafiltration—PAC/UF, coagulation/UF—CoA/UF) for the removal of selected micropollutants from river water. It was shown that the removal efficiency of benzophenones by coagulation depends on the season, while CF was moderately removed (40–70%). The removal of neutral BP by two PACs unexpectedly differed (near 40% and ˃93%), while the removal of BP-3 was excellent (>95%). PACs were not efficient for the removal of hydrophilic CF. Combined PAC/C/F/S yielded excellent removal for BP and BP-3 regardless of PAC type only when the PAC addition was followed by C/F/S, while C/F/S efficiency for CF diminished. The combination of UF with PAC or coagulant showed also high efficacy for benzophenones, but was negligible for CF removal.

## 1. Introduction

The presence of emerging microcontaminants in water bodies is a growing environmental problem because many of these microcontaminants are non-biodegradable, persistent/pseudopersistent and bioaccumulative. Some of them easily pass water treatment barriers and end up in drinking water as undesirable. River water has been widely used as a source of drinking water [1]. Examples of some studies (Table 1) showed the presence of pharmaceuticals, personal care products, pesticides, perfluoroalkyl substances, detergent degradates, flame retardants, plasticizers, polycyclic aromatic hydrocarbons etc., in river water and consequently in tap water. For most of organic micropollutants, removal obtained by conventional process, such as coagulation/sedimentation/filtration was <30% [2,3]. Using activated carbon significantly contributes to their removal but some, especially hydrophilic compounds, still remain in the water [3,4]. A better risk management is needed and therefore, it is necessary to study in more detail how these micropollutants behave in drinking water treatment. For the vast majority of the compounds, permissible concentrations in drinking water are not regulated by law.

For the purpose of this study three ubiquitous contaminants were selected: benzophenone (BP), benzophenone-3 (BP-3) and caffeine (CF). The reason for their selection is the fact that these substances are widely used and well-known anthropogenic markers from a group of pharmaceuticals and personal care products. Their limit values in waste, surface and drinking waters are not regulated, but their presence was confirmed in both river water and drinking water. BP and BP-3 were found in river water in the range from 0.002 µg/L to 44 µg/L [6,7], while CF was present in the range from 0.41 µg/L to 50 µg/L [8,9]. Lorraine and Pettigrove [10] collected samples from four water filtration plants in San Diego County, California, during August 2001 and June 2002 and reported the occurrence of BP in one out of 15 samples in a concentration of 0.26 µg/L. In the study Diaz Cruze et al. [11], BP-3 was confirmed in tap water in the range of 0.022 to 0.30 µg/L. Analysing 113 samples of drinking water in 13 cities in China revealed the occurrence of CF in 88% of the samples with median concentrations of 24.4 ng/L [12]. In a recent study, Bogunović et al. [13] confirmed the biodegradation of BP, BP-3 and CF in the Danube river water using a laboratory test filter filled with inert material, but also relevance of CF for drinking water treatment due to higher breakthrough potential through river sediment material [14]. In addition, the difference in the compounds’ hidrophylicities (logD values for BP 2.96, for BP-3 3.65 and for CF 0.28) and the molecular charge in the water solution were important for the selection. At pH 8.0 hydrophobic BP is neutral, while 89% of hydrophobic BP-3 dissociates and takes the form of a negatively charged ion. CF is neutral. This may cause different behaviour in drinking water treatment trains (DWT).

Numerous drinking water treatment installations use coagulation/flocculation/sedimentation (C/F/S) and/or powdered activated carbon (PAC) adsorption/sedimentation (PAC/S) as conventional treatment. Coagulation is efficient for natural organic matter (NOM) removal [15,16], while efficacy is low (<30%) for organic micropollutants [2]. It is known that coagulation removes large molecular-weight NOM while PAC adsorption is efficient in removing small molecular-weight NOM, as well as micropollutants. However, the data on the efficiency of these processes for emerging contaminants removal in real installations are still not systematized, while new knowledge on how NOM nature and structure influence the processes is still needed. A better understanding of the mechanisms involved could lead to improvement of the practice in the already existing installations (e.g., carbon and coagulant choice, dosing practice, etc.). The combination of these two processes, especially dosing sequences, has already been studied, but mainly for the NOM and disinfection byproducts precursor removal [15,17,18,19]. A lack of knowledge regarding the removal of emerging organic pollutants is evident. In a recent study, Campinas et al. [20] showed that high removal of organic micropollutants may be achieved by PAC/coagulation/flocculation/sedimentation (PAC/C/F/S; 65–79% removal of total-pharmaceuticals and 73–83% of total-pesticides) when 3–9 mg/L of mesoporous PAC or 20–24 mg/L of microporous PAC is added to low turbiditysurface water (≤3 NTU).Both separate PAC/S and hybrid PAC/C/F/S have the potential to be used as membrane pre-treatments or treatment alternatives to direct river water treatment by membrane.

Among the widely used and most investigated non-oxidative water treatments today is membrane filtration. Low-pressure techniques, such as microfiltration (MF) and ultrafiltration (UF) are used for the removal of suspended solids, colloids and microorganisms. For example, the technical feasibility of spiral wound ultrafiltration membranes was shown in direct eutrophic water treatment by Mierzwa et al. [21,22]. The combination of these processes with PAC or other types of sorbents is attractive for the additional removal of organic microcontaminants [23], while the combination with coagulation is attractive for natural organic matter removal [24,25]. Using the combination of adsorption onto PAC/coagulation/microfiltration by ceramic membranes, Campinas et al. [26] showed the removal of total micropollutants from surface water between 75% and the complete removal (final concentration below the limit of quantification -LOQ) of total-pesticides (with 4–18 mg/L of PAC and 2–3 mg/L Al_2_O_3_) and 82–98% for total-pharmaceuticals (with 7–18 mg/L PAC and 2–3 mg/L Al_2_O_3_). Bearing in mind an urgent need to solve the issue of emerging contaminants in drinking water, these hybrid processes seem to be attractive alternatives to the conventional techniques currently used. However, research efforts are needed to investigate their effectiveness and optimization both in lab-scale and real water treatment conditions.

The aim of this study was to preliminarily assess the efficiencies of various hybrid processes (PAC/C/F/S, PAC/UF and coagulation/UF) for the removal of ubiquitous anthropogenic micropollutants (benzophenone, benzophenone-3 and caffeine) from river water and to compare them to separate PAC/S and C/F/S under the same experimental conditions. This study is expected to help additionally understand how this reflects onto the potential of hybrid process applications in drinking water treatment for the removal of organic micropollutants. The initial concentration level of microcontaminants applied in this study (30–40 µg/L) was higher than in real river water to avoid potential interference of background compounds in the assessment of the removal. A higher concentration level also made the sample preparation step easier.

## 2. Materials and Methods

### 2.1. Coagulant, Flocculant and Powdered Activated Carbons

Coagulation/flocculation/sedimentation was performed using 1% polyaluminiumchloride (BOPAC^®^). Magnafloc^®^ LT 26 was used as a flocculant(copolymer of sodium acrylate and acrylamide with a medium degree of anionic charge [27]). Adsorption onto the PAC/sedimentation was performed using two carbons, PAC A and PAC B. PAC A is a commercial activated carbon used in the DWT, while PAC B is another, grinded type of PAC prepared for use in combination with membrane filtration. According to its supplier, PAC A has a surface area of 875 m^2^/g and a particle diameter D_50_ 15 µm. According to LeovacMaćerak [28] its mean pore radius is 20.3 Å, micropore volume determined by t-test is 0.203 cm^3^/g (27%), while total pore volume was measured as 0.745 cm^3^/g. The surface area of PAC B was 1290 m^2^/g and its particle diameter is D_50_ 4.31 µm. The mean pore radius is 16.9 Å, total pore volume 1.157 cm^3^/g and micropore volume 0.290 cm^3^/g (25%) [28].

### 2.2. Organic Solvents

For the sample preparation of organic micropollutants, three solvents—dichloromethane (≥99.8%, Ultra Resi—analyzed^®^, J.T. Baker (Mallinckrodt Baker, Inc, Phillipsburg, NJ, USA)), n-hexane (95.0%, Ultra Resi—analyzed^®^, J.T. Baker (Mallinckrodt Baker, Inc, Phillipsburg, NJ, USA)) and methanol (≥99.8%, for HPLC, Chromasolv^®^, (Honeywell, Charlotte, NC, USA)) were used. 

### 2.3. Organic Micropollutants

Investigated organic micropollutants in experiments were benzophenone, (purity ≤99%, Sigma-Aldrich, USA), benzophenone-3 (purity 98%, Sigma-Aldrich, China) and caffeine (purity >95%, Sigma-Aldrich, China).

Table 2 summarizes the physico-chemical properties of the selected compounds.

Stock solutions (5–10 mg/L) of each substance were prepared in distilled water by sonication for 3 h andfiltration was conducted through a 0.45 μm cellulose nitrate membrane filter. They were further diluted and used for spiking of the river water in experiments.

### 2.4. Water Matrix

**Figure 1:**
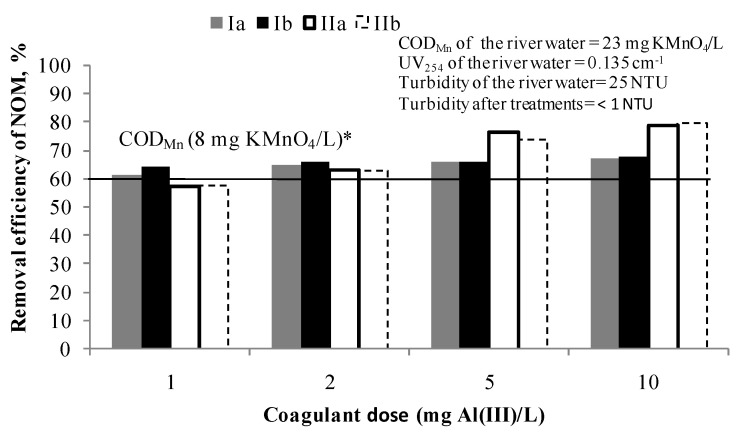
Removal efficiency of NOM by C/F/S at doses: 1, 2, 5 and 10 mg Al(III)/L. Note: Ia—removal efficiency (COD_Mn_) in experiment; Ib—removal efficiency (COD_Mn_) in experiment duplicate; IIa—removal efficiency (UV absorbance at 254 nm) in experiment; IIb—removal efficiency (UV absorbance at 254 nm) in experiment duplicate; * The line represents the removal efficiency when target value of COD_Mn_ of 8 mg KMnO_4_/L was achieved.

**Figure 2:**
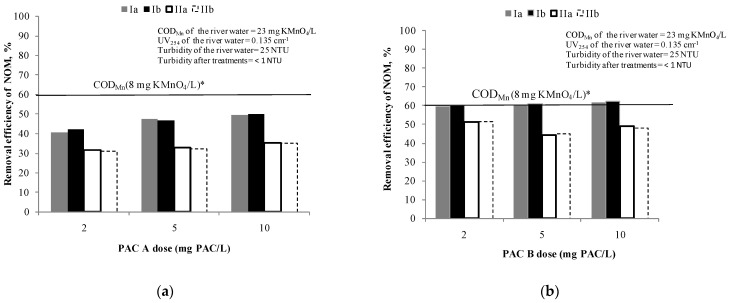
Removal efficiency of NOM by: (**a**) PAC A /S (doses: 2, 5 and 10 mg PAC/L) and (**b**) PAC B /S(doses: 2, 5 and 10 mg PAC/L). Note: Ia—removal efficiency (COD_Mn_) in experiment; Ib—removal efficiency (COD_Mn_) in experiment duplicate; IIa—removal efficiency (UV absorbance at 254 nm) in experiment; IIb—removal efficiency (UV absorbance at 254 nm) in experiment duplicate; * The line represents the removal efficiency when target value of COD_Mn_ of 8 mg KMnO_4_/L was achieved.

**Figure 3:**
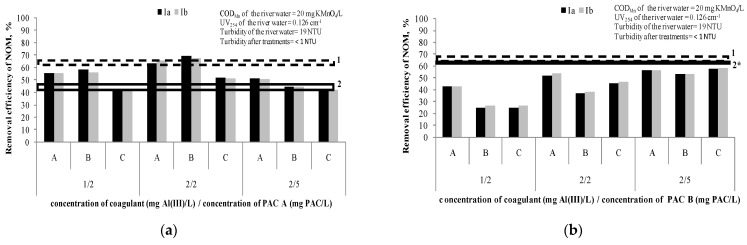
Efficiency of NOM removal using hybrid processes (A, B and C dosing sequences): (**a**) coagulant doses of 1 and 2 mg Al(III)/L and doses of PAC A 2 and 5 mg PAC/L; (**b**) coagulant doses of 1 and 2 mg Al(III)/L and doses of PAC B 2 and 5 mg PAC/L. Note: Ia—removal efficiency (COD_Mn_) in experiment; Ib—removal efficiency (COD_Mn_) in experiment duplicate; 1—Dashed line represents removal efficacy of separate C/F/S with coagulant dose range 1–5 mg Al(III)/L (63–66%); 2—Line represents removal efficacy of separate PAC A /S at doses 2 and 5 mg of PAC/L (41% and 47%, respectively); 2*—Line represents removal efficacy of separate PAC B/S at doses 2 and 5 mg of PAC/L (60% and 61%, respectively).

Samples of the Danube river water were taken upstream of the city of Novi Sad (Serbia) sewage discharge points. Only samples taken in 2017 were used for experiments presented in this paper. However, to give an overview of the main characteristics of the river water, an extended period 2015–2017 is presented in Table 3. Natural organic matter was characterized by a chemical oxygen demand test performed with potassium permanganate oxidant COD_Mn_, since this parameter is regulated by Serbian legislation (the maximum allowable value is 8 mg KMnO_4_/L) [33]. It is important to mention that the measurements of general water quality were performed only in a series of experiments used for NOM removal (chapter Section 2.5, Section 2.5.1, Section 2.5.2, Section 2.5.3) and exact values for particular samples are given later in the Figures 1–3. In experiments related to micropollutants removal those parameters were not tested.

Concentrations of the selected compounds in the river water before experiments were for BP from <0.2–0.95 µg/L (*n* = 9), BP-3 <0.5–0.62 µg/L (*n* = 7) and for CF <0.2–0.7 µg/L (*n* = 7). Their presence above LOQ values was confirmed only in 1–2 samples per compound. For the purpose of testing, such river water was spiked with a water solution of micropollutants to achieve the level of 30–40 µg/L. For each single experiment, separate C_0_ was measured and used for calculations.

### 2.5. Lab-Scale Treatments

Separate processes of C/F/S and PAC/S were first tested to find experimental conditions when the target value of COD_Mn_ is reached as defined in relevant legislation [33]. Based on the results, it was decided which dose of coagulant and PAC to apply for the removal of NOM from water in further tests of hybrid process PAC/C/F/S. They were also the basis for testing different hybrid processes for the removal of BP, BP-3 and CF: PAC/C/F/S, PAC/UF and CoA/UF. Additionally, separate PAC/S and C/F/S were tested also for BP, BP-3 and CF removal in order to compare results with hybrid process achievements. The majority of the experiments were performed in duplicate. The initial concentrations of micropollutants for the experiments involving separate PAC/S and C/F/S processes and combined PAC/C/F/S process were in the range of 30–40 μg/L, while for experiments involving membrane processes, lower initial concentrations were observed for BP-3 (3–13 µg/L), most probably due to adsorption of BP-3 on the wall of the tank (made of high-density polyethylene) used for the feed water. The experiments (C/F/S, PAC/S and combined PAC/C/F/S with PAC A were conducted in the summer season, while with PAC B (except PAC/UF and CoA/UF) the experiments were conducted in the autumn season. This is important to note since the nature of dissolved organic carbon can fluctuate [34] over the year and a direct comparison of experimental results is not possible. All the experiments related to PAC /S, C/F/S, PAC/C/F/S treatments were performed using JAR-testing apparatus, (FC6SVELP scientific). The conditions of mixing (time and speed) were typical for jar tests with the intent to be similar toa potential real drinking water treatment scenario in C/F/S treatment plants.

#### 2.5.1. C/F/S

For the removal of NOM from the water, the coagulant was dosed at 1, 2, 5 and 10 mg Al(III)/L into the samples (500 mL) and mixed at 120 rpm for 2 min. This was followed by a flocculant addition at dose 0.2 mg/L at the beginning of the slower mixing period of 26 min. After mixing, the samples were allowed to settle for 1 h, then filtered through a 0.6 µm glass fiber filter (ROTH MN 85/70).

In order to investigate the removal efficiency of the benzophenones (BPs) and CF, the selected dose of 2 mg Al(III)/L of coagulant was used as previously explained. The experiments were performed in duplicate. Experimental conditions are shown in the Table 4.

#### 2.5.2. PAC/S

For the removal of NOM from the water the adsorption test was performed by dosing the sample (500 mL) with activated carbon (2, 5 and 10 mg PAC/L) in the same way as described in 2.5.1. Initial mixing at 120 rpm for 5 min was followed by mixing at 30 rpm for 25 min. This is usually applied PAC contact time [35] After allowing the sample to settle for 1 h it was filtered through a 0.6 µm glass fiber filter (ROTH MN 85/70). In this way, it was possible to compare activated carbon efficiency with and without the coagulant addition. For the removal of BPs and CF the selected dose of PAC A/B was 2 mg PAC/L, (a dose sufficient to achieve the target criteria for drinking water [33] by PAC B and hybrid PAC A/C/F/S). All experiments were performed in duplicate. Experimental conditions are shown in Table 4.

#### 2.5.3. Hybrid Process of PAC/C/F/S

The lab-scale experiments were conducted with PAC A and PAC B in order to examine the combined effects of PAC/S and C/F/S and the influence of dosing sequences for the removal of NOM. The selected experimental conditions based on previous experiments are presented in Table 4.After mixing, the samples settled for 1 h, were filtered through a 0.6 µm glass fiber filter (ROTH MN 85/70) and analyzed. For the removal of BPs and CF by the hybrid process of PAC/C/F/S, the selected dose of coagulant was 2 mg Al(III)/L and PAC A/B was 2 mg/L since at least one of them achieve the applied target criteria for drinking water [33]. All the experiments were performed in duplicate.

#### 2.5.4. Hybrid Process of PAC/UF

The experiments were performed using the lab-scale plant (30 L/h) equipped with dizzer^®^ Lab module with Multibore^®^ 0.9, inge GmbH (7 capillaries in one fibre) and a 0.2 m^2^ membrane surface. One filtration cycle was performed for 30 min at a flux of 130 L/(m^2^h). PAC B was dosed continuously in-line (4.6 mg PAC/L) during the cycle. The total composite water sample from the whole cycle was collected and analyzed. In between the experiments, the plant was intensively backwashed, forward flushed and also additionally washed by filtration with dechlorinated tap water which did not contain the selected substances. This was performed to minimize contamination in the subsequent cycle. In addition, ultrafiltration under the same conditions of flux and cycle duration was tested separately to assess what is the contribution of sorption of micropollutants onto membrane surfaces using the same lab-scale plant without PAC application. It is important to note that the module used in the experiments was not new, but previously used in a series of experiments with dechlorinated tap water. However, the transmembrane pressure during all the experiments did not exceed 1.5 bar, which is the maximum allowable in filtration specified by the manufacturer. It ranged from 1.3–1.5 bar.

#### 2.5.5. Hybrid Process of CoA/UF

CoA/UF was performed by continuous in-line dosing of the coagulant (0.6 g Al(III)/L) to achieve a concentration of 3mgAl(III)/L using the same lab-scale plant. The coagulation/flocculation time was 30 s. Two filtration cycles and the sampling were performed in the same way as described in 2.5.4. It is important to mention that investigation of the hybrid process of adsorption onto PAC/CoA/UF had also been planned. However, due to the pump failure, this experiment was not possible to perform within an acceptable timeframe.

### 2.6. Analytical Methods

Chemical oxygen demand (COD_Mn_) was measured by the Kübel-Tiemann method [36] before and after separate C/F/S, PAC/S treatments and the hybrid processes PAC/C/F/S. The measured values presented in mgKMnO_4_/L can be easily transformed into oxidisability (mgO_2_/L) by dividing them with factor 3.95. Precision for COD_Mn_ measurement was estimated at up to 10%. NOM removal was also assessed by measuring UV absorbance at 254 nm (Shimadzu UV-1800 spectrophotometer, Japan) using a 1 cm quartz cuvette [37]. Relative standard deviation was assessed as 0.25% for 10 sample replicates. The dissolved organic carbon (DOC) content in drinking water was not used for establishment of criteria for coagulant/flocculant or PAC dose, since for this parameter there is no defined limit value [33].Conductivity, pH and turbidity of the river water were determined according to SRPSEN 27888:1993 [38], SRPSH.Z.1.111:1987 [39] and SRPSENISO7027-1:2016 [40], respectively.

Analysis of the microcontaminants was performed by the in-house developed method. The water samples (200 mL) for the analysis of BPs and CF were extracted by liquid-liquid extraction, similarly to the procedure applied by Gomez et al. [41] for analysis of BP-3 and other organic micropollutants. Hexane extraction was performed with 2 × 20 mL for BPs analysis, while for CF analysis, samples were extracted with 2 × 20 mL of dichloromethane. All the samples were dried with anhydrous sodium sulphate and evaporated to dryness under a gentle nitrogen stream. Next, they were dissolved in 0.3 mL of hexane in the case of BPs, and a 0.3 mL 1:1 dichlormethane/hexane mixture in the case of CF. Internal standards, benzophenone-d10 (>99%, Sigma Aldrich) for BPs quantitation and phenanthrene-d10 (p.aFluka) for CF quantitation, were added into the samples prior to the liquid-liquid extraction (10 μg/L).

BP, BP-3 and CF were measured by GC/MS (Agilent 7890B GC with 5977A MSD). The separation was achieved on a column Agilent J&W Scientific, HP-5MS 30 m × 0.25 mm ID × 0.25 μm. Helium was used as a carrier gas for chromatographic analysis (1 mL/min). The initial column temperature was 60 °C. After 3 min it was raised to 300 °C at the rate of 15 °C/min. The temperature of 300 °C was held for 5 min. Splitless injection was used at 250 °C. Target ions and qualifiers are presented in Table 5.

Matrix-matched calibration was performed by spiking the river water with appropriate aliquots of methanol solutions of BP, BP-3 and CF. The linearity of response was confirmed for the range of 0.2–44 μg/L for BP, 1–42 μg/L for BP-3 and for CF 1–41 μg/L in river water (R^2^ = 0.990–0.999). The limit of detection (LOD) for BP, BP-3 and CF were 0.07, 0.16 and 0.06µg/L, respectively, while corresponding limits of quantitation (LOQ) were 0.2, 0.5 and 0.2 µg/L. Precision determined as the relative standard deviation of triplicate measurements at concentration levels of 1 μg/L and 30 μg/L was ≤15% in all the cases. The bias (the quotient between the mean observed and the spiked concentration) for benzophenones was ≤5%, and ≤8% at two different concentration levels, while for CF it was 24% at a concentration level of 1 μg/L and 4% at a concentration level of 30 μg/L.

### 2.7. Removal Efficiency

The removal efficiency of the NOM (RE_NOM_) was calculated using Equation (1):

RE_NOM_ (%) = (C_0_ − C_x_)/C_0_ × 100 (1)

where C_0_ is the initial NOM concentration measured either as COD_Mn_ or UV absorbance at 254 nm in matrix without treatment, and C_x_is the NOM concentration after the treatment. All experiments were done in duplicate. Since the difference between the duplicate initial concentrations was less than 10%, the average initial concentration was compared with each concentration after the treatment.

The removal efficiency of the selected compounds by lab-scale treatments (RE_BP,BP-3,CF_) was calculated using Equation (2):

RE_BP, BP-3,CF_(%) = (C_0_−C_x_)/C_0_·100 (2)

where C_0_ is the initial concentration of compounds in matrix before the treatment and C_x_ is the concentration of compounds after different sample treatments. When the experiments were performed in duplicates, it was possible to calculate 4 removal efficiencies by comparing each initial concentration with each concentration after the treatment. In the case of experiments including CoA/UF, calculations were performed separately for each cycle related concentration.

Compounds found in samples below the LOQ were allocated values of LOQ/2. In this case, Equation (3) was used:

RE_BP,BP-3,CF_(%) = (C_0_ − LOQ/2)/C_0_ × 100 (3)

where C_0_ is the initial measured concentration in matrix before treatment and LOQ/2 is half the limit of quantitation for each compound after treatment.

## 3. Results and Discussion

### 3.1. Removal of Natural Organic Matter

#### 3.1.1. C/F/S Efficacy for the Removal of NOM

Figure 1 shows the removal efficiency of NOM after C/F/S with 1, 2, 5 and 10 mg Al(III)/L. It was calculated based on Equation (1). The efficiency of the NOM removal after C/F/S tests was determined by the chemical oxygen demand using KMnO_4_ as an oxidising agent and by measuring the UV absorbance at 254 nm.

Target quality criteria (COD_Mn_ of 8 mg KMnO_4_/L) was achieved for all applied coagulant doses and the NOM removal efficiency was 63–67% COD_Mn_. In the case of UV absorbance at 254 nm, it can be concluded that at higher doses of a coagulant the more selective the process for UV_254_ absorbing NOM compounds removal becomes (from 57% for 1 mg Al(III)/L to 80% for 10 mg Al(III)/L). Based on these results, a dose of 1 and 2 mg Al(III)/L were selected for the further testing in the hybrid processes PAC/C/F/S as sufficient to achieve the target value of COD_Mn_.

#### 3.1.2. PAC/S Efficacy for the Removal of NOM

Figure 2 show the efficiency of NOM removal using different doses of PAC A and B (from 2 mg of PAC/L to 10 mg of PAC/L), respectively.

Based on the results, none of these doses of PAC A is sufficient to achieve the applied target criteria for drinking water oxidisability [33]. However, when writing this manuscript the criterion has been changed to 12 mg KMnO_4_/L [42]. During the adsorption test with PAC A (Figure 2a), the concentration of NOM in experiment was reduced at a dose of 2 mg PAC/L by 40% and at 5 and 10 mg PAC/L around 50% in comparison to the initial NOM content. At the same time the removal efficiency of UV absorbance at 254 nm was near 30% at all carbon doses meaning that no selectivity to removal of UV_254_ absorbing compounds (mostly aromatics) can be achieved by increasing the PAC dose. Since there were no significant differences in removal by increasing the dose in applied range, the doses of 2 and 5 mg PAC/L were selected for further testing of combined PAC/C/F/S.

The PAC B (Figure 2b) proved to be more efficient than PAC A for the removal of NOM (based on COD_Mn_ it was 59–62% and based on UV absorbance at 254 nm it was 44–52%). Even with the lowest dose of PAC B of 2 mg PAC/L (removal 60%), the value of COD_Mn_ of 8 mg of KMnO_4_/L was achieved and therefore the same doses of 2 and 5 mg of PAC/L were selected for further testing of combined PAC/C/F/S. Different behaviour for COD_Mn_ and UV_254_ removal for the two types of PAC with similar volumes of micropores (25% vs. 27%) shows that a larger surface area (1290 m^2^/g) and higher total pore volume 0.630 cm^3^/g compared to commercial PAC A (surface area 875 m^2^/g; total pore volume 0.497 cm^3^/g)most probably caused the different behaviour of COD_Mn_ and UV_254_.

#### 3.1.3. Hybrid PAC/C/F/S Process Efficacy for the Removal of NOM

Figure 3 presents the removal efficiency of NOM after the hybrid processes, at different doses of coagulantand PAC A or PAC B (1 mg Al(III)/L/2 mg PAC/L; 2 mg Al(III)/L/2 mg PAC/L; 2 mg Al(III)/L/5 mg PAC/L). The applied dosing sequences (A, B and C) are explained in Table 4. We assumed that the small difference of COD_Mn_ and UV_254_ among the water samples used in comparison to the water samples used for experiments described under Section 3.1.1 and Section 3.1.2 (Figure 1 and Figure 2) are negligible.

Based on the results given in Figure 3a and taking into account the precision of measurements, it can be concluded that the hybrid processes of PAC/C/F/S with PAC A have a higher efficiency (65–69%) than separate PAC/S (41%),only when doses of 2 mg Al(III)/L and 2 mg PAC/L are applied either together or if the PAC is added after the coagulation (sequences B and A, respectively). However, it was not significantly higher than the efficiency of the separate C/F/S process (66%). These results indicate thenecessityof careful adjustment of the coagulant and adsorbent doses in a hybrid process.

In the case of the hybrid process adsorption onto PAC B and coagulation/flocculation/sedimentation (Figure 3b), it can be concluded that the hybrid process does not contribute to better removal of NOM (26–58%) compared to separate PAC/S (~60%) and separate C/F/S (63–66%). The reason is most probably the mutual interaction of the coagulant/flocculant and carbon, such as partially blocking the active sites of the PAC particles during the process of flocs formation [15]. In the study Tomaszewska et al. [15], the authors investigated the removal of humic acids and phenol from a model solution (COD_Mn_~50 mg KMnO_4_/L) by separate coagulation/sedimentation and two sequences of a hybrid process of coagulation and adsorption on PAC(firstly the PAC addition followed by the coagulant addition and simultaneous PAC addition with the coagulant). Polyaluminumchloride was applied as a coagulant at doses ranging from 1.52 to 7.62 mg of Al(III)/L, while PAC dose was 700 mg/L. It was shown that a hybrid process of successive dosing firstly with the PAC and afterward with the coagulant was the best (81–89% removal of COD_Mn_). A separate coagulation achieved 40–60% removal of COD_Mn_. This is opposite to the results in both our experiments with PAC A and PAC B. A possible reason could be a different water matrix and level of COD_Mn_ (river water vs. model water spiked with humic acid and phenol) and much higher doses of PAC used in their study [15], as well as doubled carbon contact time. Simultaneous dosing of the PAC and coagulant also gave better results than separate coagulation, but not as efficiently as the other sequence where the PAC was added first.

### 3.2. Removal of Organic Micropollutants

The removal efficiency of BPs and CF was investigated by adsorption onto PAC A and PAC B, by coagulation with coagulant, and by hybrid processes in the case of PAC A adsorption/coagulation/flocculation/sedimentation, and in the case of PAC B adsorption/coagulation/flocculation/sedimentation, PAC/UF, and CoA/UF.

#### 3.2.1. Efficiency of PAC A in Different Processes

##### Separate and Hybrid Processes of PAC/C/F/S

The removal efficiencies for separate and combined processes of PAC A/S and C/F/S are presented in Table 6. The doses of the PAC A and coagulant were 2 mg PAC/L and 2 mg Al(III)/L, respectively, while dosing sequences were: A—successive dosing of the coagulant followed by the PAC; B—simultaneous dosing of the PAC and coagulant and C—firstly dosing with the PAC followed by the coagulant (details presented in Table 4). All the experiments were conducted in the summer season but without characterisation of the general water characteristics of the specific samples used for experiments.

PAC A was effective for the removal of BP-3 (>99%) and moderately effective for BP (36 and 41%), while for CF it was not effective at the low PAC dose (2 mg/L). The high efficacy of PAC A for the removal of BP-3 can be explained by its high logD (3.65, Table 2). Similarly, in the study of Westerhoff et al. [2], the removal efficiency from surface water for BP-3 was at least 93%, while for CF the efficacy at least 70%. Westerhoff et al. [2] used much lower concentrations of the selected compounds (10–250 ng/L), higher doses of the PAC (5 mg/L), higher contact time of the PAC (4h) and also different kinds of the PAC, which all may contribute to reaching different results. We obtained an unexpected result for BP bearing in mind also relatively high logD (2.96 Table 2) and the fact that this molecule does not dissociate in water and has no charge, contrary to BP-3 which exists in water at a pH 8.0 in both neutral (11%) and negative forms (89%). One can speculate that NOM present in water could compete more effectively for adsorption sites than with partially dissociated BP-3. It is expected that the PAC charge can also play a role here. However, we could not measure this parameter in our work to be able to further discuss it.

C/F/S reduced concentrations of BP (22% to 29%), BP-3 >99%, while CF was removed 40 to 50% (Table 6). The reason for such a good BP-3 removal might also be its presence in dissociated form, which may better interact with coagulant. Removal of hydrophilic CF (log D 0.28) was higher with the C/F/S (40–50%) than with PAC/S(<11%), most probably due to the hydrophilic nature of amorphous Al hydroxide flocs [43].Nam et al. [5] discusses complicated mechanisms that occur in water treatment plants during the coagulation of natural waters: interaction with clay particles, photodegradation, and electrostatic interaction between micropollutants and coagulants. All of these affect the removal through simultaneously occurring processes of adsorption, hydrolisis and photolysis. By performing carefully designed, lab-scale studies, they showed that a significant contribution to the high removal of CF during the coagulation stage in water treatment plants (near 70%) can be provided by sunlight photolysis (lab-scale experiment with 12 h sunlight photodegradation), while coagulation in the dark removed only 20% of the compound, which is similar to the low removals observed by the other researchers for the coagulation stage. It was also shown [5] that negatively charged, hydrophilic sulfonamide-type micropollutants (C_0_ = 100 ng/L) at pH 7 can be effectively removed (~50%) from river water by coagulation using polyaluminiumchloride (30 mg/L). Westerhoff at al. [2] showed that the some hydrophobic polyaromatic hydrocarbons have high removals (60–80%), while most pharmaceuticals and personal care products have removals <25%, including CF.

In the hybrid processes PAC/C/F/S, BP was excellently removed only with dosing sequence C (≥91%), while other sequences showed efficacy between 14 and 26%, which means that the interaction of the coagulant/flocculant and adsorbent prevents the removal when the coagulant/flocculantare added first or together with carbon. Either direct competition or displacement can occur. A much better result in hybrid process C where the coagulant/flocculantare added some minutes after the carbon than in separate PAC/S, where both the pollutant and NOM are in contact with carbon 90 min can be explained by desorption prevention when the coagulant/flocculantare applied. However, this hypothesis has to be tested in future work. These findings show that different dosing sequences might be needed for NOM and microcontaminants removal. High removal efficiencies (>99%) in all dosing sequences for hybrid processes were achieved for BP-3, while CF removal was no higher than 20% but also shows low experimental repeatability. The separate roles of the coagulant and flocculant was not investigated. However, this could be a relevant topic for further research.

#### 3.2.2. Efficiency of PAC B in Different Processes

##### Separate and Hybrid Processes of PAC/C/F/S

Table 7 shows the results of the removal of BP, BP-3 and CF by PAC B/S, C/F/S and the hybrid PAC/C/F/S process. The doses of the PAC B and coagulant used in the experiments were 2 mg PAC/L and 2 mg Al(III)/L, respectively. All experiments were conducted in the autumn season but without characterisation of the general water characteristics of the specific samples used for experiments.

Based on the results shown in Table 7, it can be concluded that the highest removal efficiency of BP (93–94%) and BP-3 (95–98%) was achieved by adsorption on PAC B/sedimentation, while the removal efficiency for CF was negligible. C/F/S was not able to remove BP, while for BP-3 removal the efficiency range was 22–28%. These results are consistent with the results published in previous surveys [2,3], where removal efficacies were <20% for pharmaceuticals and personal care products, but different than the results which we obtained for the summer season (Table 6).Moreover, the C/F/S achieved considerable removal of CF of almost 70%. Significant differences between the efficiencies of the C/F/S process over the two periods were observed for BPs and CF could have resulted from the influence of seasonal variations to the content and nature of NOM. However, since we did not measure general characteristics of the water samples for experiments with micropollutants, we could only analyse official data of the Environmental Protection Agency of the Republic of Serbia from 2015–2017 (12COD_Mn_ measurements per year) [44,45,46]. The ranges of measured values for COD_Mn_ during the four seasons of 2015, 2016 and 2017 were compared and there were no significant seasonal differences among them within each year. The analysis of seasonal mean values and their confidence intervals confirmed this. In 2017 [46], when the experiments were performed, the seasonal mean values and their confidence intervals (*n* = 3) were 3.4 ± 0.4 mgO_2_/L for winter, 4.2 ± 1.8 mgO_2_ /L for spring, 4.2 ± 1.2 mgO_2_/L for summer and 3.5 ± 1.2 mgO_2_/L for autumn. Increasing the frequency of measurements would give a clearer picture. Moreover, knowledge of seasonal variations in the nature of NOM that interacts with pollutants and process materials (e.g., coagulants and adsorbents) would also contribute to clarify the relevance for water treatment efficiency similarly to the study of So et al. [34], where sophisticated technique liquid chromatography-organic carbon detection-organic nitrogen detection was used [34].

With regard to the hybrid process of PAC/C/F/S, all dosing sequences had considerable removal efficiency in the case of BPs (60–96%). However, the repeatability of experiments should be improved in some cases (e.g., sequence A and B). The sequence C had the best efficiencies for BPs (89–96%), while removal for CF was 14% and 29% in two experimental duplicates. It was shown that coagulation activity for CF (~70%) was diminished when PAC was added(8–36%), regardless of the sequence applied. Although higher efficiencies can be expected based on the specific surface of PAC B, the comparison of PACs efficiencies is not relevant, since experiments were conducted in different seasons when NOM can differ in quality.

##### Hybrid Membrane Processes

Table 8 presents the obtained results for the retention of selected compounds on the ultrafiltration membrane, as well as the efficiency of PAC/UF and CoA/UF processes for their removal from river water.

Based on the results shown in Table 8, it can be concluded that the removal for hydrophobic BPs (logD 2.96 for BP and 3.65 for BP-3) is more than 80% during UF, most probably by sorptive interactions with membranes, while hydrophilic CF (logD 0.28) is not removed. A confirmation for the sorption of BP-3 (77%) on the sulfonated polyethersulfone ultrafiltration membrane coated with an ultrathin polyimide is found in literature [47]. Yoon et al. [47] confirmed the rejection of hydrophobic organic micropollutants caused by adsorption onto UF membranes. Garcia-Ivars et al. [48] observed that highly soluble pharmaceuticals (e.g., CF) showed low rejection values (~15%) during ultrafiltration.

During the PAC/UF process, the removal efficiency of BP slightly increased to 92% from 82% (achieved by UF), while the removal of BP-3 was the same as achieved by UF (>84%). The removal of CF by the PAC/UF process was negligible. In our experiments during CoA/UF, BP removal slightly decreased in comparison to UF to 70%, while BP-3 removal was >96%. CF was not removed. Separate coagulation tests included flocculant addition and much longer contact time (fast mixing 2 min, slow mixing 25 min, and settling 60 min) than in the membrane hybrid process (30 s) and that might be the reason why separate coagulation achieved the removal of ~50%. Wray and Andrews [49] tested the efficiency of the hybrid CoA/UF process (aluminum sulfate doses of 0.5 and 15 mg/L) to remove 16 organic micropollutants (Co = 1 µg/L) from three surface waters with different organic matter contents (DOC: 2–6 mg/L). However, they performed CoA/S as a pretreatment (much longer coagulation/flocculation phase than in our case) to UF (bench scale hollow fiber polyvinylidene fluoride membrane modules ZW-500) and achieved 25% more efficient removal of certain substances (bisphenol A, estriol, sulfamethizole, naproxen and diclofenac) in relation to separate processes of coagulation and UF. The effects were dependent on water source and were not possible to be related to compound properties.

As mentioned before, the hybrid treatment of PAC/CoA/UFwas planned, but, due to technical difficulties, not performed. It is important to find the answer if coagulant addition affects PAC/UF removal, not only for hydrophilic CF but also for hydrophobic BPs.

## 4. Conclusions

Even though the hybrid process powdered activated carbon adsorption/coagulation/flocculation/sedimentation does not contribute to better removal of NOM compared to separate coagulation/flocculation/sedimentation, and only in rare cases contributes to better removal than in separate PAC adsorption/sedimentation, some interesting differences were observed for the removal of BP, BP-3 and CF. Regarding the adsorption during the PAC/S process, it is not clear if a type of activated carbon or seasonal NOM changes affected the fate of less hydrophilic neutral BP, while less hydrophilic and negative BP-3 was efficiently removed regardless of the carbon applied. Significant improvements were obtained, compared to separate processes, when adsorption was followed by C/F/S. The removal of highly hydrophilic and neutral CF was negligible by PAC/S, but separate C/F/S was 40–70% efficient, depending on the season. However, C/F/S efficiency diminished once activated carbon was added regardless of the coagulant and carbon addition sequence. The membrane hybrid process PAC/UF showed high efficiency in removing BP and BP-3 (92% and ˃84%, respectively), while CoA/UF also showed considerable removal of BP and BP-3 (on average 71% and >96% respectively). CF removal was negligible in both membrane hybrid processes. Future work should focus on clarification of the significance of the sorption of the compounds onto membrane in hybrid processes. A considerably higher number of experimental repetitions and longer lab-scale and pilot plant experiments should make it possible. Also, the hybrid membrane process where activated carbon is added together with coagulant should be tested. The work showed that characteristic of pollutants (their hydrophilicity and molecular charge) may influence the process efficiency. However, careful optimization and combination of the processes and materials has the potential to overcome difficulties in the removal of pollutants. Seasonal NOM quality fluctuations should be taken into account together with possible NOM-process material-pollutant interactions (e.g., mainly sorptive interactions both with PAC and flocs formed during coagulation). Similarly, the possibility to preserve separate coagulation/flocculation activity for hydrophilic CF removal in hybrid processes should be further investigated.

## Figures and Tables

**Table 1:** Examples of the studies related to emerging microcontaminants in river and drinking water.

Drinking Water Treatments	Substances Detected in Source -River Water	Substances Detected in Treated Drinking Water	References
Clarification/disinfection/sand/granular activated carbon filtration/disinfection	Compounds detected in at least 75% samples in concentration below 1.2µg/L: polycyclic musk fragrances, pharmaceuticals and their degradates, insect repellents, organophosphorus flame retardants and plasticizers, polycyclic aromatic hydrocarbons, solvent tetrachloroethene, cosmetics like triethyl citrate and benzophenone	21 compounds detected in concentration <0.5 µg/L in at least one sample, despite general decrease in average concentration of compounds from source to treated waters, reason might be incomplete degradation or removal through the treatment process. The average percent removal of these compounds by granular activated carbon filtration was 53%; by disinfection was 32%, and by clarification 15%	Stackelberg et al. [3]
Coagulation/sedimentation/sand filtration/postchlorination	12 out of 14 tested organic micropollutants were detected. Among the detected compounds, metoprolol, carbamazepine, acetaminophen, caffeine, naproxen, sulfamethoxazole, sulfamethazine, and ibuprofen had higher detection frequencies (>50%)	11 compounds were detected, and the highest average concentration was observed for metoprolol, around 35 ng/L. Diverse removal efficiencies (6–100%) for the detected compounds were obtained. Caffeine, acetaminophen, carbamazepine and 2,4-dichlorophenoxy acetic acid were effectively removed (>80%), while the most recalcitrant was metoprolol (removal 6%).	Nam et al. [5]
Pre-ozonation/flocculation-coagulation/decantation/ sand filtration/post-ozonation/granular activated carbon filtration	Among 60 compounds tested 31 were detected with individual median concentrations below 10 ng/L for all the compounds except caffeine (64.1 ng/L).	17 compounds were detected. The most detected were perfluoroalkyl substances, terbuthylazine, atrazine and their degradation products desethyl-terbuthylazine and desethyl- atrazine, simazine, caffeine and imidacloprid.	Borrull et al. [4]

**Table 2:** Physico-chemical properties of the selected compounds.

Compound	Abbreviations	CAS ^a^	Use ^b^	MW ^b,c^ (g/mol)	pKa ^b^	LogD ^d^ (at pH 7.4)	Charge (at pH 7.4)
Benzophenone	BP	119-61-9	Flavoring agent, UV filter	182	n.a	2.96	0
Benzophenone-3	BP-3	131-57-7	UV filter	228	7.1	3.65	[0,-] ^e^
Caffeine	CF	58-08-2	Stimulant	194	0.82 ^f^;14	0.28	[0] ^e^

^a^ Chemical abstract service registry number; ^b^ PubChem, [29]; ^c^ Molecular weight; ^d^ ChemSpider; [30]; ^e^ Rossner et al. [31]; ^f^ Onaga Medina et al. [32].

**Table 3:** Characteristics of the river matrix.

Parameter	Unit	Range	Median Value	Number of Samples
COD_Mn_	mg KMnO_4_/L	11.1–23.2	20.0	12
pH		6.4–8.1	7.97	17
Eh	μS/cm	333–623	377	17
UV_254_	cm^−1^	0.106–0.135	0.125	5
Turbidity	NTU	13.7–25.2	13.7	5

COD_Mn_—chemical oxygen demand using KMnO_4_ oxidant agent; Eh—conductivity.

**Table 4:** Experimental conditions for separate C/F/S and PAC /S and hybrid process of PAC/C/F/S.

Process	PAC Type	PAC Doses (mg/L)		Coagulant Doses (mg Al(III)/L) ^a^		Mixing Conditions	Parameters Analysed	
		1	2	1	2		1	2
C/F/S	n/a	n/a	n/a	1, 2, 5 and 10	2	120 rpm 2 min, 30 rpm for 26 min	COD_Mn_ and UV_254_	BP, BP-3 and CF
PAC/S	A and B	2, 5 and 10	2	n/a	n/a	120 rpm 5 min, 30 rpm for 25 min		
PAC/C/F/S	A and B	2 and 5	2	1, 2, 5	2	A—Successive dosing of coagulant (120 rpm 2 min) followed by flocculant addition at the beginning of the slow mixing phase (30 rpm, 1 min) and subsequent PAC addition (30 rpm for additional 25 min)		
						B—Simultaneous PAC and coagulant dosing (120 rpm 2 min) followed by addition of flocculant (30 rpm 25min)		
						C—Successive dosing of firstly PAC (120 rpm 5 min), followed by coagulant (120 rpm 2 min) and afterwards flocculant (30 rpm 25 min)		

^a^ in all coagulation experiments flocculant was dosed 0.2 mg/L; 1—experiments related to NOM removal; 2—experiments related to organic micropollutants removal; n/a—not applicable.

**Table 5:** Target ions and qualifiers.

Compound	Target Ion (m/z)	Qualifier (m/z)
Benzophenone	105	182; 77
Benzophenone-3	151	228; 227; 77
Benzophenone-d10	110	192; 82
Caffeine	194	109; 82
Phenanthrene-d10	188	160; 80

**Table 6:** Removal efficiency of BP, BP-3 and CF by PAC/S, C/F/S and combined PAC/C/F/S.

Processes	BP	BP-3	CF
PACn/S(2 mg PAC/L)			
Removal efficiency, %	36	>99	11
	41	>99	−2
C/F/S (2 mg Al(III)/L)			
Removal efficiency, %	22	>99	50
	28	>99	43
	22	>99	48
	29	>99	40
PAC/C/F/S(2 mg PAC A/L/2 mg Al(III)/L)			
Dosing sequence A			
Removal efficiency, %	19	>99	17
	26	>99	5
Dosing sequence B			
Removal efficiency, %	18	>99	4
	25	>99	−11
	14	>99	6
	21	>99	−8
Dosing sequence C			
Removal efficiency, %	92	>99	20
	93	>99	8
	91	>99	10
	92	>99	−3

**Table 7:** Removal efficiency of BP, BP-3 and CF by PAC B/S, C/F/S and hybrid PAC/C/F/S process.

Processes	BP	BP-3	CF
PAC B/S (2 mg PAC/L)			
Removal efficiency, %	94	>98	−1
	94	>98	7
	93	95	*
	94	95	*
C/F/S (2 mg Al(III)/L))			
Removal efficiency, %	4	22	70
	6	24	67
	1	27	*
	3	28	*
PAC/C/F/S (2 mg PAC B/L/2 mg Al(III)/L)			
Dosing sequence A			
Removal efficiency, %	75	59	36
	75	60	17
	86	87	*
	87	87	*
Dosing sequence B			
Removal efficiency, %	86	87	17
	87	87	8
	87	62	*
	87	63	*
Dosing sequence C			
Removal efficiency, %	89	95	14
	89	96	29
	90	96	*
	91	96	*

* In the case of CF only one initial concentration was used, which is why only two RE are shown.

**Table 8:** Removal efficiency of BPs and CF by hybrid membrane processes. Note—experiment was performed in summer season.

Processes	C_0_ (BP) 25–31 µg/L	C_0_ (BP-3) 3.2–13 µg/L	C_0_ (CF) 31–36 µg/L
	Removal Efficiency, %		
UF	82	>84	1
PAC/UF	92	>84	12
CoA/UF			
I experiment *	73	>96	12
II experiment *	69	>96	4

* The experiments were conducted in the same water sample.

## Data Availability

Data are contained within the article.
